# Immunomodulatory properties of extracellular vesicles isolated from bone marrow of patients with neuroblastoma: role of PD-L1 and HLA-G

**DOI:** 10.3389/fimmu.2024.1469771

**Published:** 2024-10-24

**Authors:** Danilo Marimpietri, Maria Valeria Corrias, Gino Tripodi, Roberto Gramignoli, Irma Airoldi, Fabio Morandi

**Affiliations:** ^1^ UOSD Laboratorio di Terapie Cellulari, IRCCS Istituto Giannina Gaslini, Genova, Italy; ^2^ UOSD Terapie Sperimentali in Oncologia, IRCCS Istituto Giannina Gaslini, Genova, Italy; ^3^ UOC Servizio di Immunoematologia e Medicina Trasfusionale, IRCCS Istituto Giannina Gaslini, Genova, Italy

**Keywords:** neuroblastoma, extracellular vesicles, HLA-G, PD-L1, bone marrow

## Abstract

**Introduction:**

Extracellular vesicles (EVs) can be released by any cell and are crucial for cell-to-cell communications. EVs have been characterized in patients with solid and hematological tumors, where they play an important role in tumor progression and metastasis. EVs may express different surface proteins derived from the parental cells, including immunomodulatory molecules, such as HLA-G and PDL1.

**Methods:**

We isolated EV from bone marrow (BM) samples of patients with Neuroblastoma (NB) and healthy controls and we analyzed the expression of CD56, GD2 and immune checkpoints on EV by flow cytometry. Next, we analyzed the function of T cells in vitro in the presence or absence of NB patients' BM-derived EV, in terms of proliferation and cytokine production. Finally, we analyzed the correlation between the expression of immune checkpoints on EV and the clinical outcome of patients.

**Results:**

We found a higher expression of CD56 on EVs derived from BM of patients with NB than in those from healthy donors (HD). However, CD56 expression was not dependent on BM infiltration of NB cells. Moreover, the analysis of GD2 expression revealed that only a small fraction of EVs was released by infiltrating NB cells, whereas the majority may derive from BM-resident cells. BM-derived EVs from NB patients display a higher expression of HLA-G and PD-L1 than those derived from HD. Nonetheless, such EVs are able to modulate T cell immune responses. We measured a robust response, in vitro, towards a common bacterial antigen, including the release of GM-CSF and proinflammatory cytokines, like IFN-a and IL-6, from mononuclear cells. Some of these immunomodulatory features are dependent on the expression of HLA-G and PD-L1, whereas others may rely on other mechanism(s). Finally, a high expression of CD56, HLA-G and PD-L1 on BM-derived EVs may represent a good prognostic factor.

**Conclusions:**

We described the presence of HLA-G and PDL1-bearing EVs in the BM of NB patients, which may represent a mechanism performed by resident BM cells to counteract the inflammation occurring in the BM microenvironment of NB patients.

## 1 Introduction

Neuroblastoma (NB) is the most common extracranial solid tumor of childhood, with an incidence of approximately 10 cases per million individuals with an age below 15 years (1). The primary tumor is derived from neural crest progenitor cells, and displays genetic and biological features which are crucial for the clinical fate of the tumor (2). Indeed, several parameters, including age at diagnosis, histologic category, tumor differentiation, the MYCN oncogene status, chromosome 11q status, and DNA ploidy are relevant for the clinical outcome of patients (3). NB patients are currently stratified by the International Neuroblastoma Risk Group Staging System (INRG-SS) into low risk (L1-stage), intermediate risk (L2-stage) and high risk (M-stage) (3). High-risk NB patients represent half of total cases and display a 50% overall survival, in spite of aggressive treatments (3). In M-stage patients, infiltration of metastatic NB cells in the bone marrow (BM) represents the worst prognostic factor for these children (4). Malignant cells infiltrating the BM express different immune checkpoints which are involved in the inhibition of anti-tumor immune responses (5–7). HLA-G is a non-classical HLA-Ib molecule exerting immunomodulatory properties through the interaction with inhibitory receptors on T lymphocytes and NK cells (8). Such a non-polymorphic HLA can be detected with seven different isoforms as surface molecules or soluble moiety, with limited expression and secretion in NB cell lines or primary tumors (9). Nonetheless, BM-infiltrating NB cells express high levels of HLA-G on their surface and secreted in the BM microenvironment high levels of sHLA-G, found to correlate directly with the stage of the disease (5, 10). Another mediator recently recognized as relevant in oncological disorders and NB is Programmed cell Death-1 (PD-1). PD-1 is an inhibitory receptor expressed mainly by T lymphocytes, but also activated NK cells (11, 12). PD-1 is expressed on T lymphocytes in the late phase of activation and, upon interaction with specific ligands, it may trigger the inhibition of several intracellular pathways started by kinases (13). Two different ligands of PD-1, namely PD-L1 and PD-L2, can be detected on antigen presenting cells (mostly PD-L2) and other cells in healthy tissues (PD-L1) (14, 15). Notably, PD-L1 expression can be induced on NB cell lines and BM-infiltrating metastatic NB cells following stimulation with IFN-γ. Thus, the PD-1/PD-L1 axis may play a role in the NB-mediated immune suppression (6). Indeed, in preclinical models, the blockade of PD-1/PD-L1 interaction increased therapeutic efficacy and phase I clinical trials are currently ongoing using anti-PD-1 or anti-PD-L1 monoclonal antibodies (16, 17). Growing interest has been raised in biological vehicles for such important molecular mediators. Indeed, tumor-derived extracellular vesicles (EVs) have been recognized as main actors in tumor progression, formation of metastatic niche and inhibition of anti-tumor immune response (18–21). EVs isolated from plasma of NB patients have been shown to vehiculate proteic cargos different from healthy subject vesicles, with an increased expression of proteins belonging to tumor-associated pathways, like nucleolin and NCAM (22). Furthermore, other factors vehiculated by those EVs modulated immune response (23, 24) and are involved in the onset of therapy resistance (25). Collectively, several studies have recently supported the role of EVs in the progression of NB (26). Recently, a direct immunosuppressive role played by EVs released by BM cells of NB patients has been elucidated, in connection with high expression of adenosinergic ectoenzymes (CD38, CD39, CD203a, and CD73) generating an immunosuppressive molecule such as adenosine (ADO) (27). To further elucidate such immune evasive capacity and specifically the role of EVs and molecules vehiculated by such soluble mediators, we i) analyzed vesicles isolated from BM of NB patients in comparison with healthy subjects; ii) assessed HLA-G, PD-1 and PD-L1 expression on such EVs; iii) evaluated the contribution of HLA-G and PD-1/PD-L1 axis on immune-regulatory properties of such EVs.

## 2 Materials and methods

### 2.1 Patients

Twenty-nine (29) samples of BM aspirates were collected from NB patients at diagnosis (9 L-stage, 16 M-stage, 2 MS-stage and 2 not defined) for diagnostic purposes from December 2010 to August 2018. Written informed consent for research use of samples and clinical data was obtained by the legal guardians. An analog cohort of BM aspirates were donated for research use by 15 healthy donors (HD), selected according to the Transplant Unit Clinical Protocol of Hematology at the IRCCS San Martino-IST in Genoa, following a written informed consent at the time of donation. The study design was approved by the IRCCS Istituto Giannina Gaslini Ethics Committee (14/3/2008) in conformity with the principles of the Declaration of Helsinki. Demographic, biological and clinical features of patients are summarized in **Table 1**. BM infiltration by metastatic NB cells was assessed by morphological analyses of 6 May-Grunwald Giemsa-stained smears of each BM aspirate. Positivity was graded by a semi-quantitative score (1+: infiltration <10%; 2+: infiltration between 10 and 30%; 3+: infiltration > 30% with respect to resident mononuclear cells).

**Table 1:** Demographic, biological and clinical features of the 29 NB patients.

N	Sex	Age (months)	Stage	BM infiltration	MYCN status	Relapse	EFS (years)	State at follow-up	OS (years)
1	Female	72	M	2	single copy	No	6,4	Alive	6,4
2	Male	43	M	2	MYCN gain	Yes	0,4	Alive	3,7
3	Female	26	M	3	MYCN gain	No	9,4	Alive	9,4
4	Female	17	M	3	amplified	Yes	0,9	Dead	1,5
5	Female	36	M	3	MYCN gain	Yes	2,0	Dead	3,0
6	Female	24	M	3	ND	Yes	1,3	Dead	1,5
7	Female	3	M	1	Amplified	No	2,4	Alive	2,4
8	Male	1	L2	0	single copy	No	4,8	Alive	4,8
9	Female	45	ND	0	ND	No	3,3	Alive	3,3
10	Female	11	M	1	single copy	No	1,1	Alive	1,1
11	Male	39	M	2	ND	Lost			
12	Female	4	MS	0	single copy	No	4,7	Alive	4,7
13	Female	17	L2	0	single copy	No	4,5	Alive	4,5
14	Female	39	L2	0	ND	Lost			
15	Female	140	M	1	single copy	Yes	3,1	Alive	4,8
16	Female	34	L2	0	single copy	No	0,3	Alive	0,3
17	Female	21	M	2	amplified	Yes	0,1	Dead	3,5
18	Male	14	M	1	amplified	No	1,7	Alive	1,7
19	Male	34	M	2	single copy	No	3,4	Alive	3,4
20	Male	45	L2	0	single copy	No	1,3	Alive	1,3
21	Female	51	M	3	single copy	Yes	2,2	Dead	2,7
22	Male	21	M	1	ND	Lost			
23	Male	4	L2	0	single copy	No	3,2	Alive	3,2
24	Female	30	M	1	MYCN gain	No	2,6	Alive	2,6
25	Female	6	L2	0	single copy	No	3,9	Alive	3,9
26	Female	30	L2	0	single copy	No	2,4	Alive	2,4
27	Male	12	L2	ND	ND	Lost			
28	Female	24	ND	ND	ND	Lost			
29	Male	32	M	ND	ND	Lost			

### 2.2 Isolation and characterization of EVs

BM aspirates were centrifuged at 3000 x g for 15 minutes at 4°C to obtain BM plasma, preserved at -80°C in small aliquots (300 μl) until use. After thawing, EVs were characterized in terms of size and number in a cohort of subjects (NB, n=14; HD, n=10) and then isolated, as reported (27). Briefly, 300 μl of each sample was diluted (1:3) in PBS and centrifuged at 3000 x g for 15 minutes at 4°C to remove platelets and large cell debris. The supernatants were collected in a suitable centrifugation tube and, on a small aliquot from these samples, EVs size and concentration were determined via a nanoparticle tracking analysis (NTA) using NanoSight NS300 (Malvern Panalytical Ltd, Malvern, UK) equipped with NTA 2.3 analytical software and a 532 nm laser (28). Then, supernatants were centrifuged at 20000 x g for 1 hour at 4°C in a fixed-angle rotor, and EVs were suspended in MACS buffer, composed by PBS/EDTA (Miltenyi Biotec, Bergisch Gladbach, Germany) with 0.5% BSA (Sigma Aldrich, St. Louis, Missouri, USA) or in RPMI (Euroclone, Milan, Italy). In a few NB samples (n=8) EVs were characterized in terms of size and yield before and after their enrichment. The whole procedure was performed under a laminar flow cabinet to preserve the sterility of EV samples, later used in *in vitro* functional studies on cultured cells.

### 2.3 Flow cytometric analysis

The expression of the immune checkpoint inhibitors on EVs was evaluated by flow cytometry using the following mouse monoclonal antibodies (mAbs, all purchased from BioLegend. San Diego, California, USA): PE-conjugated anti-HLA-G (clone #87G), Alexa Fluor 488 conjugated anti PD-1 (clone NAT105), PE/Cyanine 7-conjugated anti-PD-L1 (clone 29E2A3). The expression of CD56 and GD2 was assessed using APC-conjugated anti-CD56 (clone AF12-7H3, Miltenyi Biotec) and PE-conjugated anti-GD2 (clone 14G2a, BioLegend), respectively. To investigate the cellular origin of EV, the following mAbs were used: FITC-conjugated anti-CD45 (clone HI30, BD), PE-conjugated anti-CD34 (clone AC136, Miltenyi Biotec), APC-conjugated anti-HLA-DR (clone G46-6, BD), PE-CF594-conjugated anti-CD3 (clone UCHT-1, BD), FITC-conjugated anti-CD19 (clone HIB19, BD), APC-conjugated anti-CD90 (clone 5E10, BD), PE-conjugated anti-CD105 (clone 266, BD) and PC5-conjugated anti-CD14 (clone A07765, Beckman Coulter). Samples were incubated in MACS buffer with specific mAbs for 20 minutes in the dark at 4°C, then diluted with 1 ml of MACS buffer and centrifuged at 20000 x g for 1 hour at 4°C. EVs were then suspended in 200 µl MACS buffer and subjected to flow cytometric analysis using a Gallios cytometer and Kaluza software (Beckman Coulter, Brea, California, USA). Results were expressed as percentage of positive EV or mean relative of fluorescence intensity (MRFI), calculated as mean fluorescence generated by specific mAb/mean fluorescence using irrelevant isotype-matched mAb.

### 2.4 Functional studies

To investigate the immune modulation exerted by BM-derived EVs isolated from NB patients, functional experiments were set up as follows. Buffy-coat preparations (BC) were obtained from 3 healthy blood donors at IRCCS San Martino after informed consent. Total mononuclear cells (MNC) were isolated by Ficoll/Hypaque density gradient (Cytiva, Marlborough, USA) following centrifugation at 2000 x g for 30 minutes at room temperature and subsequent washing steps. MNCs were seeded in flat-bottom 96 well plates at 1x10^6^ cells/well in RPMI (Euroclone) supplemented with 5% human AB serum (obtained from normal donors enrolled by our Institute). After quantification, each EV preparation was sub-divided into three aliquots: one left untreated, one treated with anti-HLA-G blocking mAb (clone 87G, pure functional grade, Miltenyi Biotec) and one with anti-PD-L1 blocking mAb (clone 2340D, R&D Systems, Minneapolis, Minnesota, USA). Each aliquot was incubated for 30 minutes at room temperature (RT), before addition to the seeded MNC in a ratio of 200:1 (200x10^6^ EVs/well). Each EV preparation was isolated from a pool of three BM samples or three different NB patients, to minimize the variability and to generate a sufficient amount of EVs for functional studies. In some wells, EVs were not added (untreated control). MNC were incubated overnight at 37°C, 5% CO2, in the presence or absence of EVs. EVs isolated from HD were not included in functional studies, since insufficient in quantity. The volume of BM plasma collected from HDs was contained to limit risks to volunteers and for ethical reasons.

#### 2.4.1 IFN-γ secretion assay

The effects of NB patients’ BM-derived EVs from on IFN-γ secretion were evaluated by using the Rapid Cytokine Inspector (RCI CD4/CD8, Miltenyi Biotec), following manufacturer’s instructions. MNC were incubated overnight as described above, and stimulated with 1 μg/ml of Staphylococcus Enterotoxin B (SEB, Sigma Aldrich). SEB served as a superantigen activating T lymphocytes, thus triggering T cell proliferation and secretion of IFN-γ and other cytokines. After incubation of MNC at 37°C, 5% CO2 for 2 hours, Brefeldin A was added to each well and incubation continued for an additional 4 hours. Next, 80 μl of supernatant were removed and 50 μl of staining mix (including anti-IFN-γ mAb) were added to each well. After 10 minutes of incubation at room temperature, equal volume of fixative and permeabilizing solutions were subsequently added and incubated for 20 and 10 minutes, respectively. Finally, MACS buffer was added and samples centrifuged at 300 x g for 5 minutes at 4°C. Supernatants were then carefully removed, and 200 μl of MACS buffer were added in each well. Cells were then analyzed by the MACS Quant 10 flow cytometer analyzer, using the automated “Express Mode” (Miltenyi Biotec) to detect IFN-γ production by intracellular staining of both CD4 and CD8 T cell populations. Results were expressed as percentage of IFN-γ+ producing cells among CD3+CD4+ or CD3+CD8+ T lymphocytes.

#### 2.4.2 Proliferation assay

T cell proliferation was assessed by flow cytometry using CellTrace™ CFSE Cell Proliferation Kit (Thermo Fisher Scientific, Waltham, Massachusetts, USA). Briefly, an aliquot of MNC preparation was suspended in RPMI and stained with 2 μM Carboxyfluorescein diacetate N-succinimidyl ester (CFSE) for 15 minutes at 37°C. Next, RPMI supplemented with 20% FBS (Thermo Fisher, Waltham, Massachusetts, USA) was added, cells were centrifuged at 300 x g for 10 minutes and suspended in RPMI containing 5% human AB serum. After overnight incubation in the presence or absence of EVs (as described above), MNC were stimulated with SEB (1 μg/ml). In some wells, SEB was not added (negative control). Plate was incubated at 37°C, 5% CO2, for 6 days. Supernatants were then collected and stored at -20°C while cells after washing were stained with anti-CD3 PC7 (Beckman Coulter), anti-CD4 APC and anti-CD8 PE (Miltenyi Biotec) for 20 minutes at room temperature. After washing, cells were suspended in MACS buffer and subjected to flow cytometric analysis using Gallios Cytometer and Kaluza software (Beckman Coulter). Results are expressed as % of proliferating CD3+CD4+ and CD3+CD8+ T cells (as witnessed by CFSE dilution).

#### 2.4.3 Cytokine secretion

The concentration of different cytokines was measured on the supernatants collected from the proliferation assay and stored at -20°C until use, by using the MACSPlex Cytokine 12 kit (Miltenyi Biotec), following manufacturer’s protocol. This assay allows to determine the concentration of GM-CSF, IFN-α, IFN-γ, IL-2, IL-4, IL-5, IL-6, IL-9, IL-10, IL-12p70, IL-17A and TNF-α in a 96 well filter plate. Briefly, supernatants were thawed and centrifuged to remove particulates at 10000 x g for 10 minutes at 4°C. The assay was carried out in 96 well filter plate after a washing step with MACSPlex Buffer (by centrifugation using an adapter at 300 x g for 3 minutes). Samples, standards and negative controls (RPMI containing 5% human AB serum) were seeded (50 μl/well) and MACSPlex capture beads added and incubated on an orbital shaker for 2 hours at room temperature. After a washing step, MACSPlex Cytokine 12 Detection Reagent was added and the plate was incubated in an orbital shaker for one additional hour. The plate was then washed twice and 200 μl of MACSPlex Buffer was added to each well before sample acquisition using MACS Quant 10 Analyzer. Samples were acquired using the “Express Mode” specific to this assay (Miltenyi Biotec). Cytokine concentration in the samples is expressed as pg/ml.

### 2.5 Statistical analysis

Statistical analysis was performed using Prism software, version 5.03 (GraphPad Software Inc.). Data distribution was analyzed using D’Agostino and Pearson omnibus normality test. Student’s t-test or Mann-Whitney test were used to compare data sets, depending on data distribution. Log-rank Test and Gehan-Breslow-Wilcoxon Test were performed to compare patients’ survival. Differences were considered significant when p value was < 0.05. Receiver operating characteristic (ROC) curve analysis was performed using MedCalc (MedCalc Software Ltd, Ostend, Belgium). Results are reported as median ± standard error (SE) or standard deviation (SD).

## 3 Results

### 3.1 Characterization of BM-derived EVs

**Figure 1:**
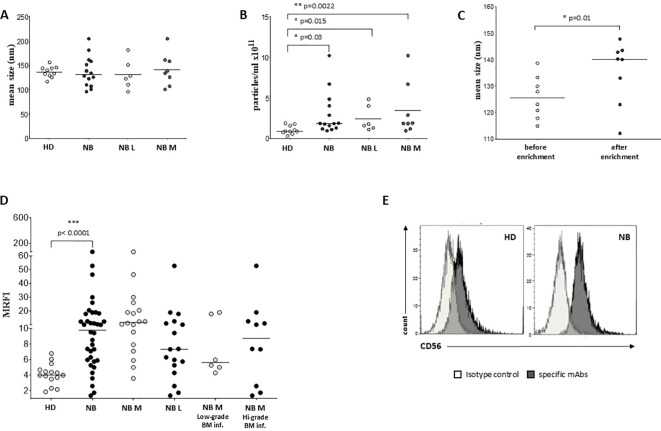
Characterization and phenotypical analysis of EVs in BM samples. The size **(A)** and concentration **(B)** of EVs has been analyzed in BM samples from healthy controls (white dots) and NB patients (black dots), and in L-stage (light grey dots) or M-stage (dark grey dots) NB patients. The results are expressed as nm and particles/ml x 10^11^, respectively. **(C)** The size of EVs has been also analyzed in samples from NB patients before (white dots) and after (black dots) EVs enrichment. **(D)** The expression of CD56 has been analyzed on EVs isolated from BM samples of i) healthy controls (white dots) and NB patients (black dots), ii) M-stage (grey dots) and L-stage (dark grey dots) NB patients and iii) M-stage NB patients with low (grey dots) and high (dark grey dots) BM infiltration of NB cells. The results are expressed as MRFI. Horizontal bars indicated medians. Statistically significant differences are indicated. **(E)** A representative staining for CD56 is shown. Grey peaks indicated staining with isotype controls, whereas black peaks indicated staining with specific mAbs.

Firstly, we determined the concentration and size of EVs in BM plasma samples collected from 10 HD and 14 NB patients (6 at L2-stage and 8 at M-stage) and by Nano Tracking Analysis (NTA). As shown in **Figure 1A**, EV size was similar between NB (median ± SE, 137.2 ± 31.4 nm) and HD (136.6 ± 11.57 nm). Minor differences were measured between M-stage (141.3 ± 33.42 nm) and L-stage (131.6 ± 30.58 nm) NB patients. Furthermore, the amount of EVs was significantly higher in BM plasma samples from NB patients (3 ± 2.65x10^11^/ml) than HD (1 ± 0.52x10^11^/ml, p=0.03) (**Figure 1B**). Indeed, both the number of EVs from L-stage (2.43 ± 1.58x10^11^/ml) or M-stage (3.43 ± 3.28x10^11^/ml) patients were significantly higher compared to HD (p=0.015 and p=0.0022, respectively), although no significant differences were measured among NB patients (**Figure 1B**). Since L-stage patients commonly do not have BM-infiltrating NB cells, we hypothesize that EVs found in the BM of NB patients do not derive exclusively from neuroblasts. Furthermore, to assess whether the enrichment of EVs through centrifugation may alter the mean size of EVs, we performed NTA on samples from a cohort of NB patients (n=8) before and after isolation. As shown in **Figure 1C**, the mean size of EVs was higher after the enrichment (mean ± SD, 135.3 ± 4.2 nm) than before (125.8 ± 8.0 nm, p=0.01). These data support the hypothesis that large EVs were enriched from BM plasma samples after centrifugation.

### 3.2 Phenotypic analysis of BM-derived EVs

**Figure 2:**
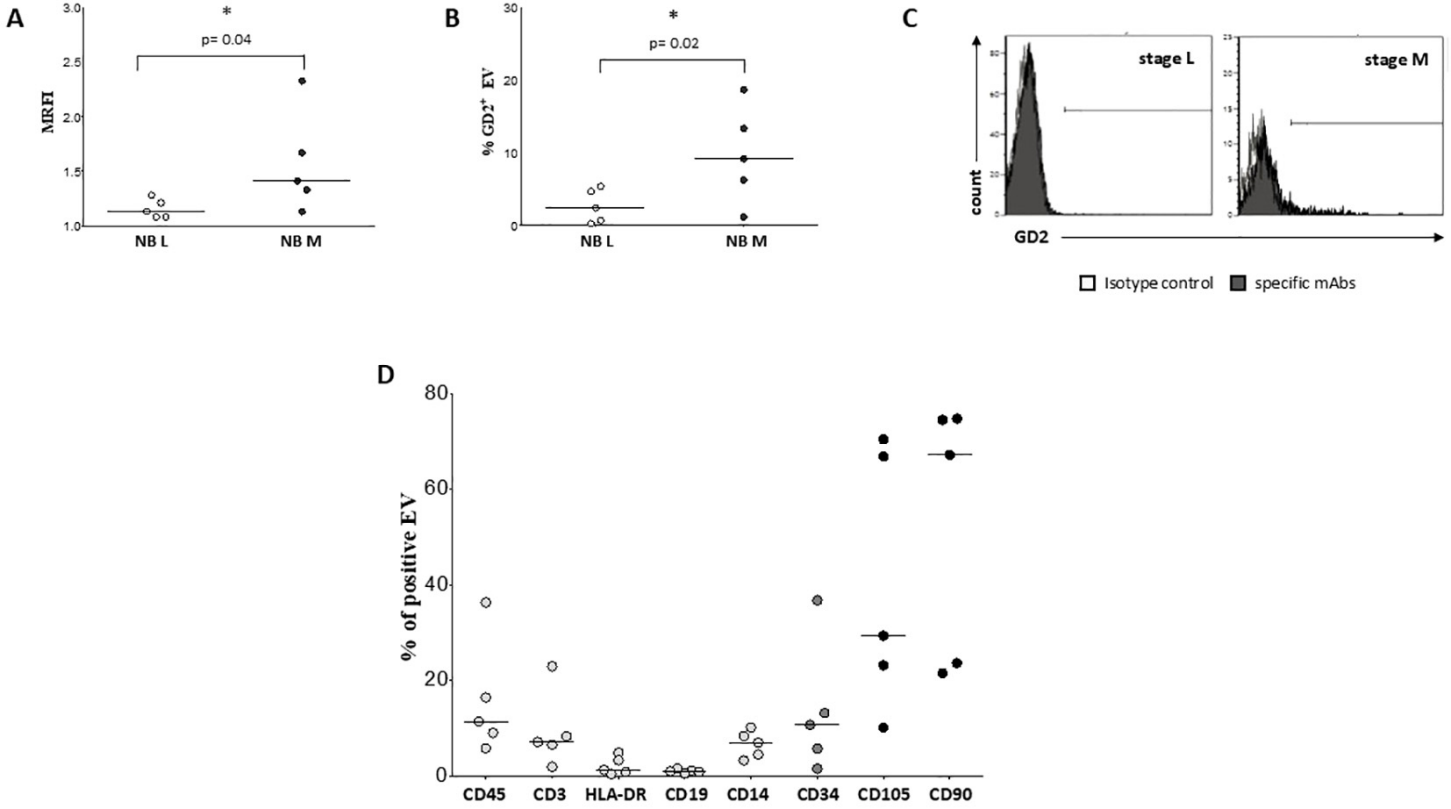
Characterization and phenotypical analysis of EVs in BM samples. The expression of GD2 has been analyzed on EVs isolated from BM samples of L-stage (white dots) or M-stage (black dots) NB patients. The results are expressed as MRFI **(A)** and % of GD2^+^ EVs **(B)**, respectively. Horizontal bars indicated means. Statistically significant differences are indicated. A representative staining for GD2 is shown in **(C)**. Grey peaks indicated staining with isotype controls, whereas black peaks indicated staining with specific mAbs. **(D)** shows the expression of CD45, CD3, HLA-DR, CD19, CD14, CD34, CD105 and CD90 on EV isolated from 5 BM samples of NB patients. The results are expressed as % of positive EV. Horizontal bars indicated medians.

**Figure 3:**
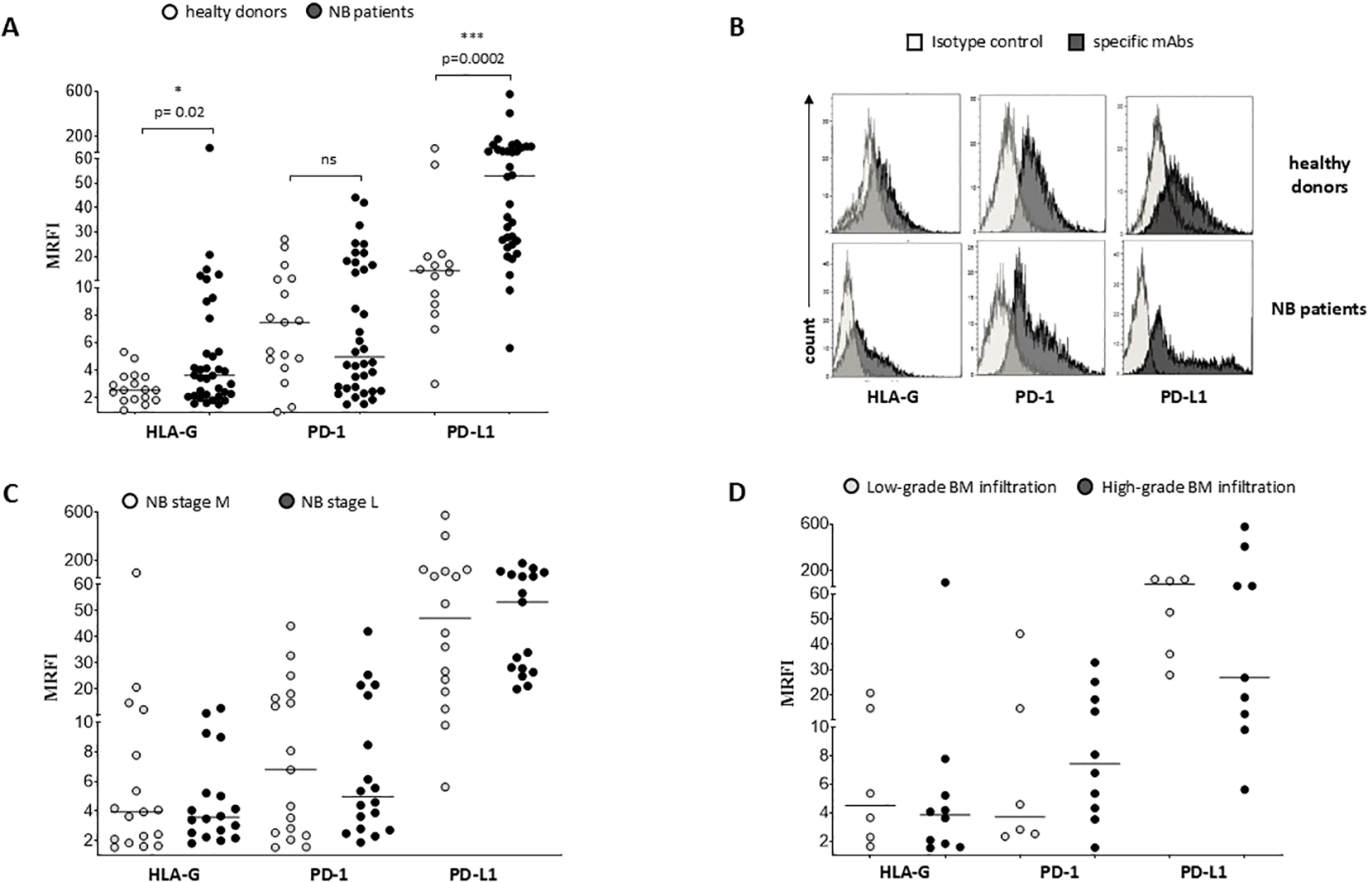
Phenotypical analysis of EVs in BM samples. The expression of HLA-G, PD-1 and PD-L1 has been analyzed on EVs isolated from BM samples of i) healthy controls (white dots) and NB patients (black dots) **(A)**, ii) M-stage (grey dots) and L-stage (dark grey dots) NB patients **(C)** and iii) M-stage NB patients with low (grey dots) and high (dark grey dots) BM infiltration of NB cells **(D)**. The results are expressed as MRFI. Horizontal bars indicated medians. Statistically significant differences are indicated. A representative staining is shown in **(B)**. ns: not significant.

**Figure 4:**
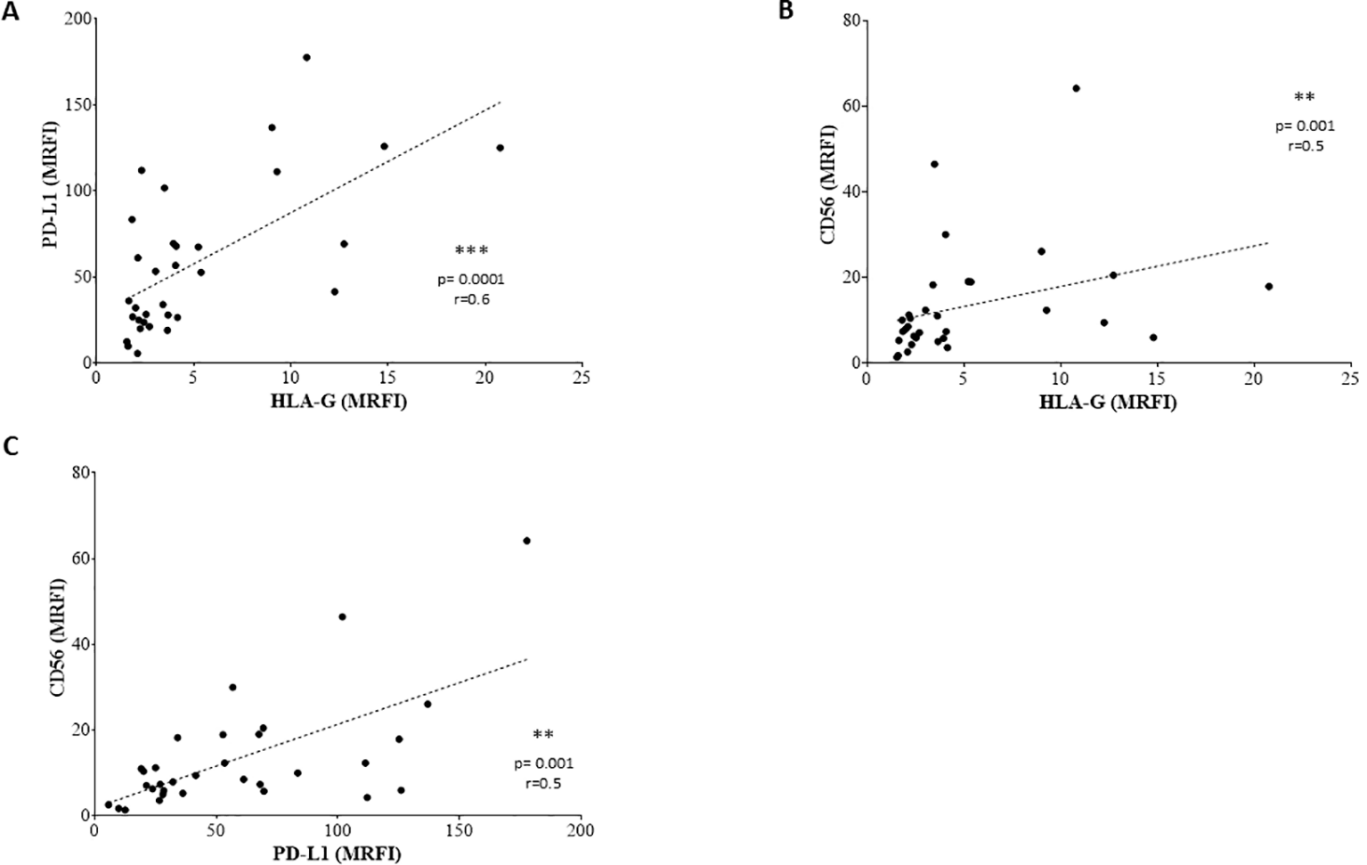
Correlations between the expression of surface molecules. Correlations are shown in **(A)** (PD-L1 vs HLA-G), **(B)** (CD56 vs HLA-G) and **(C)** (CD56 vs PD-L1). Lines indicated linear regression. Statistically significant differences are indicated.

We exposed EV preparations to flow cytometric characterization for selective membrane mediators. First, we determined the expression of CD56 on EVs isolated from BM plasma samples of NB patients and healthy donors. CD56 was chosen as NB-associated marker, although its expression can be detected also on NK cells, activated T cells, monocytes, and dendritic cells (29). As shown in **Figure 1D**, the expression of CD56 was significantly lower in EVs isolated from 17 HD (MRFI, median ± SE:4.01 ± 0.35) than in those from 29 NB patients (20 at M and 9 at L stage; 9.79 ± 2.35, p<0.0001). CD56 expression on EVs was similar in both M-stage (n=20, 7.39 ± 2.63) and L-stage (n=9, 11.25 ± 3.89) NB patients. We next compared the expression of CD56 on EVs isolated from M-stage NB patients with low (grade 1) or intermediate/high (grade 2-3) infiltration of NB cells in the BM, as assessed by cytomorphological analysis of BM smears (see M&M for details). No significant difference was measured in such cohorts of patients (5.64 ± 2.81 and 8.75 ± 4.78, respectively in low- or high-grade infiltration). Collectively, these data suggested that CD56 may be derived from resident BM cell populations rather than infiltrating NB cells. A representative staining for CD56 on EVs is shown in **Figure 1E**. Thus, we investigated the expression of disialoganglioside GD2, another NB-associated antigen, on EVs isolated from a cohort of NB patients (5 L-stage and 5 M-stage). As shown in **Figure 2A**, the expression of GD2 was significantly higher in EVs from M-stage (MRFI mean ± SD, 1.57 ± 0.20) than in L-stage (1.15 ± 0.08, p=0.04) NB patients. Since GD2 was not expressed on the whole EVs population but in a small fraction, we also analyzed the percentage of GD2^+^ EVs. As shown in **Figure 2B**, the percentage of GD2^+^ EVs was higher in M-stage (% GD2^+^ EVs, mean ± SD, 9.77 ± 2.99) than in L-stage (2.70 ± 1.02, p=0.02) NB patients. A representative staining for GD2 on EVs is shown in **Figure 2C**. The extremely low GD2 expression on EVs isolated from BM of NB patients, in particular in L-stage subjects where it was virtually undetectable, suggested such vesicles were mainly derived from BM-resident cells rather than BM-infiltrating NB cells. Thus, we investigated the expression of different markers for leukocytes (CD45), T lymphocytes (CD3), B lymphocytes (CD19), monocytes (CD14), hemopoietic stem cells (CD34), antigen-presenting cells (HLA-DR), and mesenchymal stromal cells (CD105 and CD90). As shown in **Figure 2D**, the percentage of CD45^+^ EV was low (% of positive EV, range 5.79-36.34, median 11.41). The majority of these EVs were CD3^+^ (range 1.96-22.92, median 7.11) or CD14^+^ (range 3.25-10.15, median), whereas CD19^+^ (range 0.53-1.56, median 0.96) and HLA-DR^+^ (range 0.37-4.92, median 1.29) EVs were low to absent. CD34 antigen was also present on these EVs (range 1.53-36.75, median 10.71). The most expressed markers on EVs were those derived from mesenchymal stromal cells (MSC): CD105 (range 10.12-70.47, median 29.36) and CD90 (range 21.53-74.79, median 67.2). Collectively, these data suggested that EVs are partially released by mature leukocytes and hemopoietic stem cells, but MSCs are the major releaser of EVs in the BM microenvironment. We expanded our analyses on additional, critical immune checkpoint inhibitors such as HLA-G, PD-1 and PD-L1. As shown in **Figure 3A**, the expression of HLA-G and PD-L1 was significantly higher in EVs isolated from NB samples (MRFI, median ± SE: 3.65 ± 2.68, and 53.06 ± 19.51, respectively) than in controls (2.58 ± 0.27 and 14.23 ± 6.60, respectively). By contrast, the expression of PD-1 was similar in NB patients and HD (4.98 ± 1.91 and 7.50 ± 1.68, respectively). A representative cytofluorimetric analysis is shown in **Figure 3D**. The expression of HLA-G, PD-1, and PD-L1 was similar when we compared M-stage (3.95 ± 5.00, 5.36 ± 2.71 and 56.97 ± 35.22, respectively) and L-stage (3.41 ± 0.93, 4.41 ± 3.09 and 43.73 ± 13.10, respectively) NB patients (**Figure 3B**). As shown in **Figure 3C**, no significant differences were detected either in M-stage patients with low- or high NB infiltration rates regarding the expression of HLA-G (MRFI median ± SE, 4.52 ± 3.2 vs 3.86 ± 9.49), PD-1 (3.71 ± 6.75 vs 7.44 ± 3.29) and PD-L1 (82.38 ± 18.75 vs 26.89 ± 69.85). Since HLA-G and PD-L1 are immune checkpoints described in tumor cells, we checked whether their expressions on BM-derived EVs isolated from NB patients are mutually associated. As shown in **Figure 4A**, HLA-G and PD-L1 expression significantly correlated on EVs isolated from NB patients (r=0.6, p=0.0001). Similar results were obtained by analyzing the correlation between CD56 and HLA-G (**Figure 4B**; r=0.5, p=0.001) and between CD56 and PD-L1 (**Figure 4C**; r=0.5, p=0.001). Collectively, these results may suggest a common origin for BM-derived EVs. Furthermore, all these data suggest that BM-derived EVs from NB patients are mainly released from BM resident cells rather than from BM-infiltrating NB cells. Thus, since CD56 may represent an activation marker on immune cell populations, BM-derived CD56+ EVs from NB patients may be derived from activated cell subsets among BM-resident cells, witnessing a high inflammatory state in the BM microenvironment of NB patients which is lacking in healthy subjects. A representative plot of distribution size of EVs and phenotypical analysis is shown in **Supplementary Figure 1**.

### 3.3 Effect of BM-derived EVs from NB patients on T cell proliferation and IFN-γ secretion

**Figure 5:**
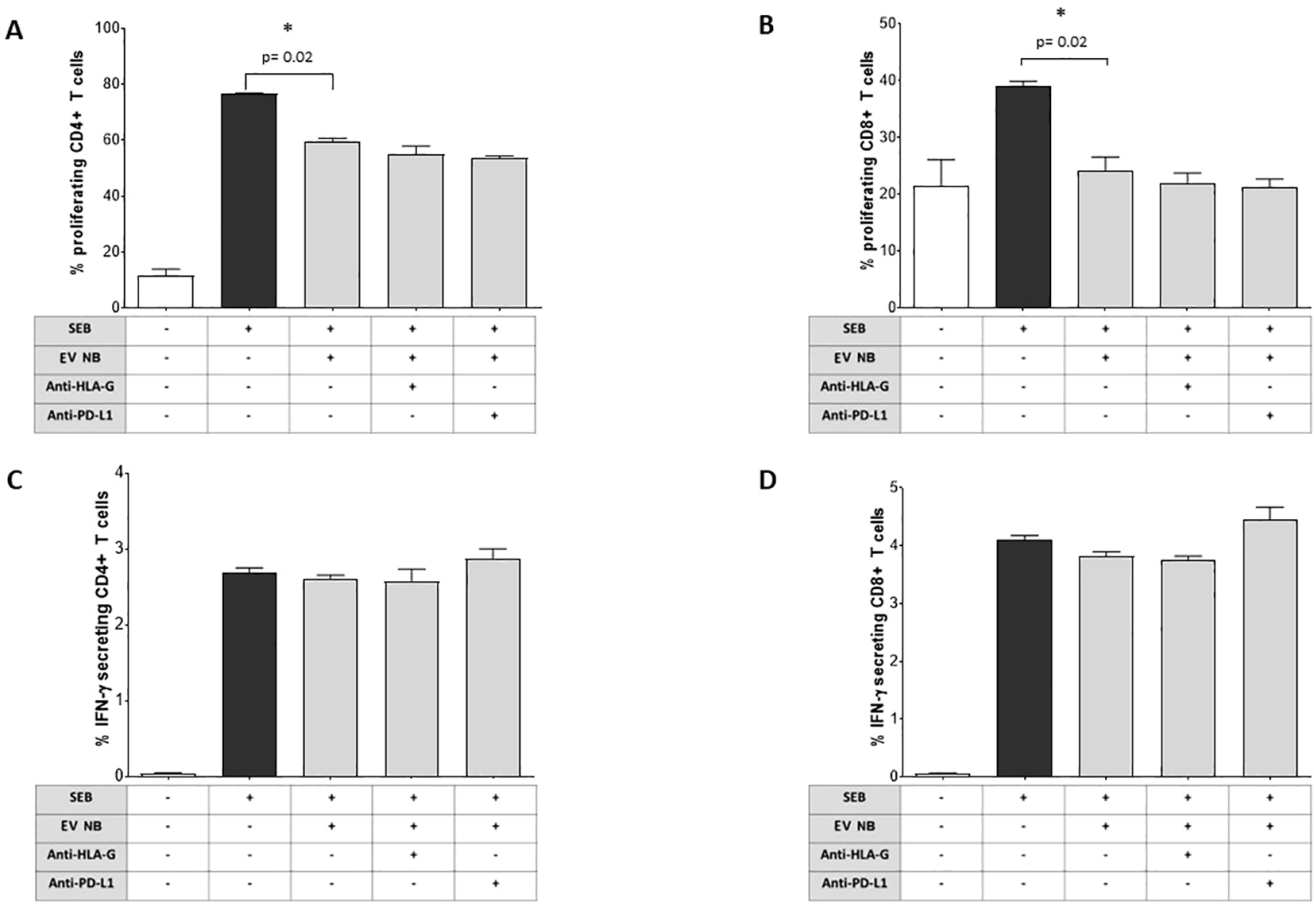
Immunoregulatory functions of NB patients’ BM-derived EVs. The proliferation of CD4 **(A)** or CD8 **(B)** T cells from normal donors (either unstimulated or stimulated with SEB) was assessed by flow cytometry in the presence or absence of NB patients’ BM-derived EVs and in the presence or absence of blocking antibodies against HLA-G or PD-L1. The results are expressed as median+SE of the % of proliferating cells (as witnessed by CFSE dilution). In the same experimental conditions, the intracellular IFN-γ secretion was assessed by flow cytometry in CD4^+^ **(C)** or CD8^+^ **(D)** T cells. The results are expressed as median+SE of the % of IFN-γ^+^ cells. Statistically significant differences are indicated.

We analyzed whether BM-derived EVs isolated from NB patients affect the proliferation of CD4^+^ and CD8^+^ T lymphocytes, previously induced by SEB. As shown in **Figures 5A, B**, the percentage of proliferating CD4^+^ increased in the presence of SEB (% of cells, median ± SE: unstimulated 10.3 ± 2.2; SEB 76.5 ± 0.21) as well as in and CD8^+^ T lymphocytes (unstimulated 18.40 ± 4.60; SEB 39.40 ± 0.96). However, the presence of EVs dampened the proliferation of both CD4^+^ (59.3 ± 1.37, p=0.02) and CD8^+^ (22.9 ± 2.37, p=0.02) T lymphocytes. Of note, blocking antibodies directed against HLA-G or PD-L1 were not effective in restoring T cell proliferation, suggesting the vesicle effect was not related to HLA-G or PD-1/PD-L1 pathways. A representative gating strategy panel is shown in **Supplementary Figure 2**. Next, we evaluated whether BM-derived EVs from NB patients were able to modulate T cell immune response against bacterial antigen SEB. Total MNC from healthy subjects were incubated in the presence or absence of EVs, and then stimulated with SEB. CD4^+^ and CD8^+^ T cell subsets were then analyzed for the intracellular IFN-γ production. As shown in **Figures 5C, D**, CD4^+^ and CD8^+^ T lymphocytes efficiently produced IFN-γ in response to SEB (% of cells, median ± SE: unstimulated 0.035 ± 0.01; SEB-stimulated 2.7 ± 0.06 and unstimulated 0.06 ± 0.01; SEB-stimulated 4.11 ± 0.08, respectively). The presence of NB-derived EVs did not alter the production of IFN-γ either in CD4^+^ (2.55 ± 0.05) or in CD8^+^ (3.89 ± 0.07) T cell subsets.

### 3.4 Effect of BM-derived EVs from NB patients on cytokine release from MNC stimulated with SEB

**Figure 6:**
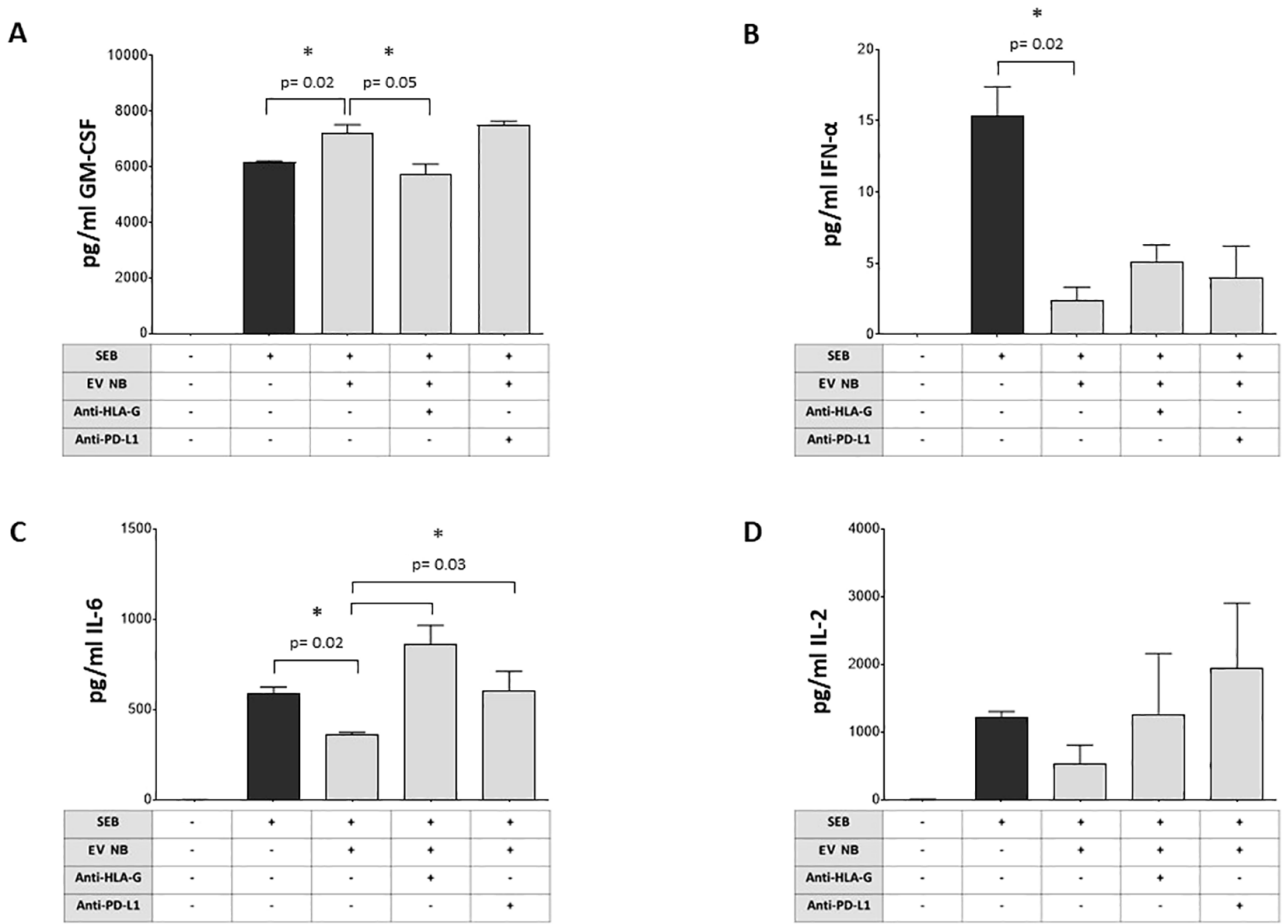
Modulation of cytokine production by NB patients’ BM-derived EVs. The secretion of different cytokines (**A** GM-CSF, **B** INF-α, **C** IL-6, **D** IL-2) by MNC from normal donors (either unstimulated or stimulated with SEB) was assessed by flow cytometry in the presence or absence of NB patients’ BM-derived EVs and in the presence or absence of blocking antibodies against HLA-G or PD-L1. The results are expressed as pg/ml (median+SE). Statistically significant differences are indicated.

We next investigated the ability of BM-derived EVs from NB patients to modulate the release of 12 pro- or anti-inflammatory cytokines by SEB-stimulated MNC. As shown in **Figure 6A**, the bacterial enterotoxin B actively induces the secretion of GM-CSF by MNC (median ± SE: unstimulated 0 ± 0.02 pg/ml; SEB-stimulated 6154 ± 49.2 pg/ml). The presence of NB-derived EVs significantly increased GM-CSF secretion (7223 ± 273.6 pg/ml, p=0.02, panel A). Such a secretion was significantly inhibited by anti-HLA-G blocking mAb (5578 ± 335 pg/ml, p=0.05), but not in the presence of anti-PD-L1 blocking mAb (7558 ± 131.4 pg/ml). Bacterial enterotoxin also induced also the secretion of IFN-α (**Figure 6B**) (unstimulated 0 ± 0.002 pg/ml; SEB-stimulated 15.35 ± 2.02 pg/ml). Once again, such a secretion was significantly and strongly dampened in the presence of NB-derived EVs (1.8 ± 0.89 pg/ml, p=0.02), with no significant effects when blocking mAbs against HLA-G (4.1 ± 1.22 pg/ml) or PD-L1 (4 ± 2.1 pg/ml) were added (**Figure 4B**). Another soluble mediator released in high quantity in the presence of SEB is IL-6 (**Figure 6C**) (unstimulated 0.35 ± 0.04 pg/ml; SEB-stimulated 617.2 ± 35.16 pg/ml). Similarly, the presence of NB-derived EVs significantly inhibited IL-6 secretion (359.4 ± 9.24 pg/ml, p=0.02). IL-6 release was significantly restored by the exposure to blocking mAbs against HLA-G (892.5 ± 105.1 pg/ml, p=0.03) or PD-L1 (574.3 ± 103.8 pg/ml, p=0.03) (**Figure 6C**). Another interleukin presenting a similar pattern was IL-2: we measured a low basal secretion equal to 1.15 ± 2.1 pg/ml in unstimulated cells, and one thousand times higher in the presence of bacterial endotoxin (1297 ± 810.5 pg/ml). NB-derived EVs inhibited IL-2 secretion (383.3 ± 271.7 pg/ml) and the supplementation of blocking antibodies partially restored the secretion, although these differences were not significant (**Figure 6D**). Finally, the production and secretion of other soluble mediators such as IL-4, IL-10, TNF-α, IL-5, IL-9, IL-12p70, and IL-17A were induced by bacterial enterotoxin, but no modulation was observed by exposure to NB-derived EVs. We were unable to determine the effects on IFN-γ secretion, since the concentration was above the limit of detection of the assay in all experimental conditions (data not shown).

### 3.5 High expression of CD56, HLA-G, PD-1 and PD-L1 and prognosis of NB patients

**Figure 7:**
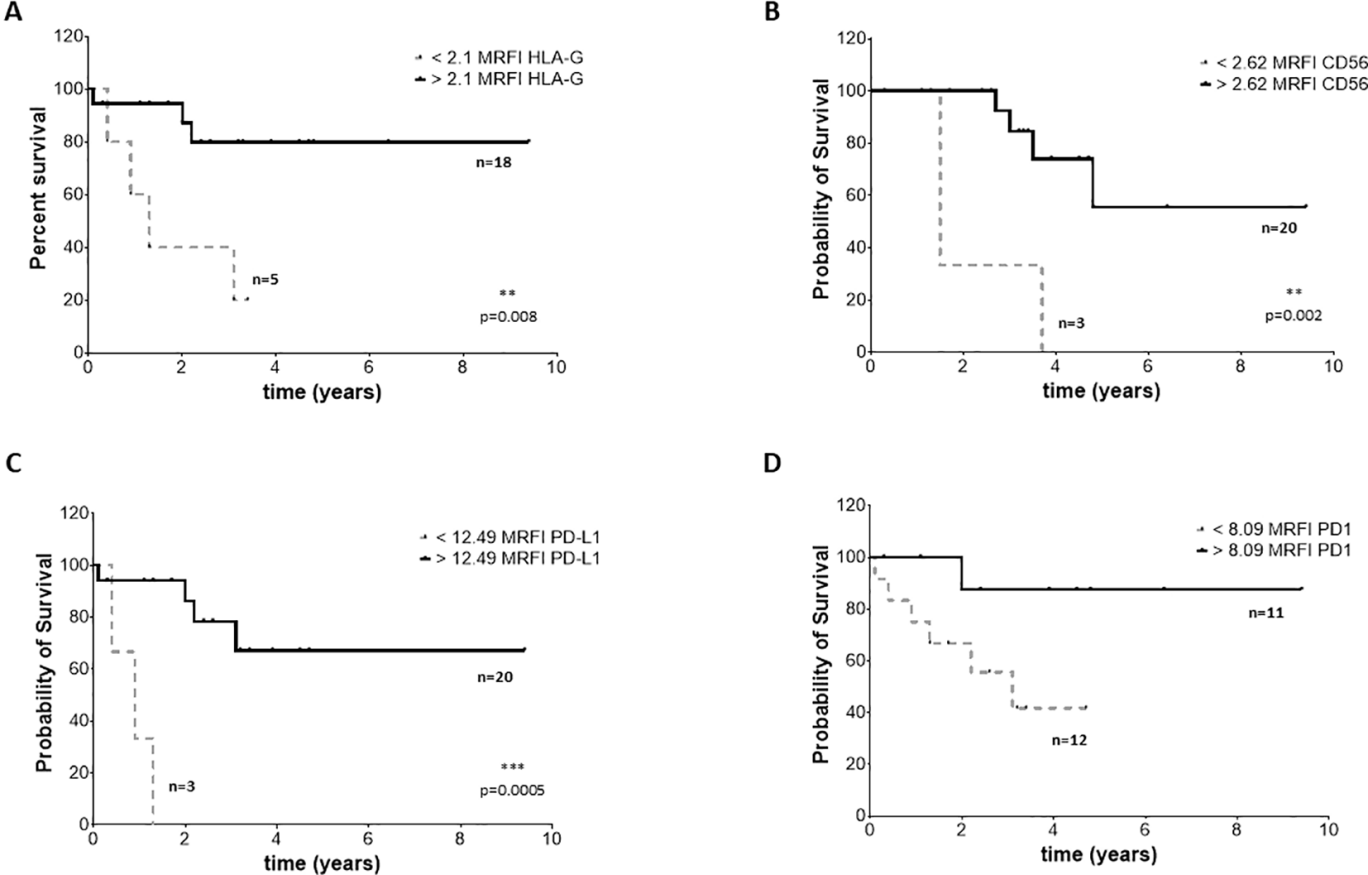
EFS survival of NB patients. Kaplan Meier curves represent EFS of patients with expression of HLA-G **(A)**, CD56 **(B)**, PD-L1 **(C)** and PD-1 **(D)** above (black continuous lines) or below (grey dotted lines) the cut-off levels. Statistically significant differences are indicated.

**Figure 8:**
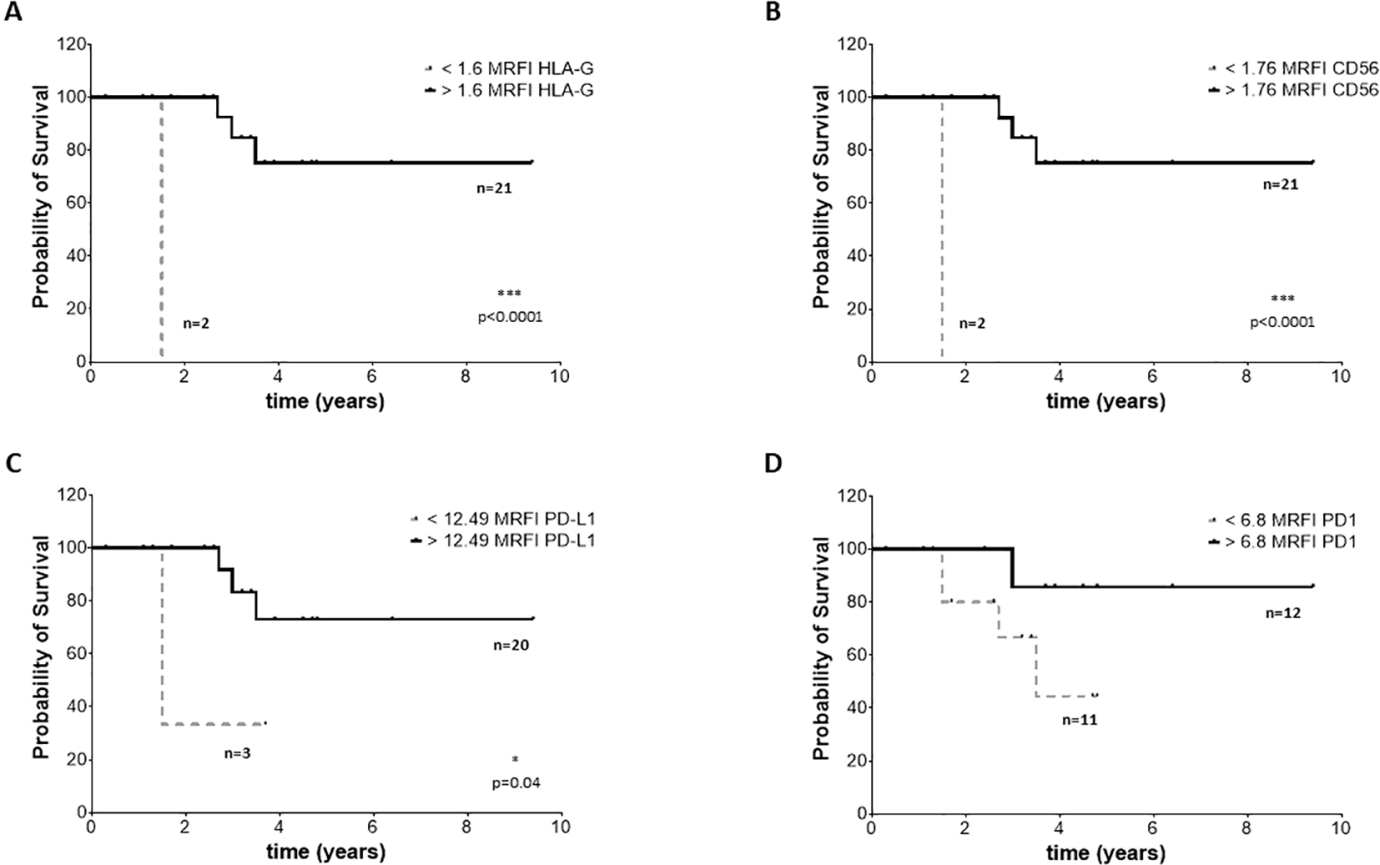
OS survival of NB patients. Kaplan Meier curves represent OS of patients with expression of HLA-G **(A)**, CD56 **(B)**, PD-L1 **(C)** and PD-1 **(D)** above (black continuous lines) or below (grey dotted lines) the cut-off levels. Statistically significant differences are indicated.

To assess whether the phenotype of BM-derived EVs may impact the clinical outcome, we selected 23 NB patients (14 M-stage, 7 L-stage, and 2 MS-stage) with available data at follow-up regarding event-free survival (EFS) and overall survival (OS). We performed an ROC curve analysis to calculate the cut-off MRFI values for CD56, HLA-G, PD-1, and PD-L1 able to discriminate patients with different prognoses. We obtained similar results for all 4 molecular signatures, with a slight difference for OS. Specifically, we measured MRFI for CD56 equal to 2.62 in EFS and 1.76 as OS; 2.1 and 1.61 for HLA-G, respectively for EFS and OS; 8.1 and 6.8 for PD-1. PD-L1 had the same result for EFS and OS (12.5). Such values were used to plot the survival curves of NB patients with MRFI values on EVs above or below the cut-off values for each molecule. Complete data of ROC analyses are shown in **Supplementary Figure 3**. Patients with MRFI values of HLA-G on BM-derived EVs above the cut-off value display a better EFS (**Figure 7A**, Hazard Ratio 13,16, 95% CI of ratio 1,940 to 89,22) and OS (**Figure 8A**, Hazard Ratio 23361, 95% CI of ratio 224,1 to 2,436x10^6^) than NB patients with MRFI values below the cut-off (p=0.008 and p<0.0001, respectively). Similar results were obtained for CD56 (EFS: p=0.002, **Figure 7B**, Hazard Ratio 51,64, 95% CI of ratio 4 to 666,8; OS: p<0.0001, **Figure 8B**, Hazard Ratio 23361, 95% CI of ratio 224,1 to 2,43x10^6^) and PD-L1 (EFS: p=0.0005, **Figure 7C**, Hazard Ratio 323,5, 95% CI of ratio 16,42 to 6373; OS p=0.008, **Figure 8C**, Hazard Ratio 17,13, 95% CI of ratio 1,064 to 276,0) expression. No significant difference in survival probability was found when the cut-off level for PD-1 expression was used (**Figures 7**, **8D**). Finally, we analyzed the number of control samples with MRFI values above the cut-off levels. The majority of control samples display MRFI values above the cut-off levels calculated for EFS (CD56 12/15, HLA-G 10/15, PD-L1 8/15, PD-1 6/15) and OS (CD56 15/15, HLA-G 13/15, PD-L1 8/15, PD-1 9/15) (data not shown).

## 4 Discussion

The important and longly underestimated role of EVs in tumor progression has gained recognition over the last few years, both for solid and hematological tumors (21, 30). Consequently, EVs have been recently proposed as tumor biomarkers for diagnostic purposes as well as possible targets for novel therapeutic strategies (31, 32). Several studies have addressed the role of EVs in the inhibition of anti-tumor immune response, in the formation of tumor metastasis and, more importantly, in the resistance to drugs in NB (26). Neoplastic cell-derived vesicles can express a variety of surface proteins, derived from the parental cells. Among them, ligands for inhibitory receptors and immune checkpoints may be relevant, since their presence on tumor-derived EVs may generate a protective halo, supporting tumor cell evasion from immune recognition. Indeed, effector cells, like T lymphocytes and NK cells are likely impeded in recognizing and eliminating neoplastic cells, shielded by secreted EVs equipped with a specific arsenal. Indeed, the presence of HLA-G-bearing EVs and their effect on T lymphocytes has been described in tumors (33, 34) and consequently proposed as a marker for tumor progression (35). Similarly, PD-L1-expressing EVs have been detected in several tumors (36–42), representing a prognostic marker for the clinical outcome as well as for the response to therapy (36–39, 43). The current study moved from such a hypothesis and elucidated the large presence of EVs bearing HLA-G and PD-L1 in samples collected from patients affected by NB. We successfully isolated extracellular vesicles from BM plasma samples withdrawn by NB patients and we detected CD56, a marker of immune cell activation, along with high amount of immune-modulatory mediators such as HLA-G, PD-1 and PD-L1. The level of expression in patients with NB was significantly higher than the amount detected in EVs equally isolated from healthy subjects. The surface expression of immune mediators was independent of the stage of NB disease, and among patients with high-risk classification, we measured independence from the degree of BM infiltration by NB metastatic cells. These findings strongly support the tenet that the BM-derived EVs are mainly secreted by BM resident cells rather than from the BM-infiltrating NB cells. In particular, our data suggested that EVs in the BM microenvironment are mainly released by MSC, with a smaller contribution from HSC and mature leukocytes. A further support for this conclusion comes from the finding that GD2, a NB-associated antigen, is expressed at very low levels, and only on a small fraction of BM-derived EVs. Furthermore, the higher the expression of CD56, HLA-G, or PD-L1 on EVs, the better the outcome of the patient. However, a multivariate analysis by the Cox-regression model, which is required to confirm the prognostic value of these observations, did not reach the statistical significance, due to the limited number of samples analyzed (data not shown). By examining the effects of the BM-derived EVs isolated from NB patients on T cells and MNC immune response we found no direct effect on the production of IFN-γ by CD4^+^ or CD8^+^ T cells, whereas they significantly dampened proliferation of both T cell subsets. The inhibition of T cell proliferation, however, is independent from HLA-G or PD-L1 expression, suggesting that inhibition was likely mediated by other mechanisms. In this context, the contribution of different molecules to the immune escape of NB cells has been reported, including IDO (22) and adenosinergic ectoenzymes (27). Patients-derived EVs enhance the secretion of GM-CSF by activated MNC, mainly through the expression of HLA-G and inhibit the secretion of IL-6 through the expression of both HLA-G and PD-L1. BM-derived EVs collected from NB patients also seem to modulate the MNC secretion of IL-2 and IFN-α by MNC. Notably, it has been reported that GM-CSF may modulate immune response through the activation of tumor-associated neutrophils and macrophages and up-regulate PD-L1 expression on the latter cells (44, 45). IL-6 may be a malevolent player in anti-tumor immune response, since it may promote survival and proliferation of tumor cells and metastatic spread. However, IL-6 may also support T cell activation, proliferation and survival and boost cytotoxic function and anti-tumor activities (46). Taken together, all these findings suggest that BM-derived EVs may positively impact NB patients’ survival through an immunomodulatory effect on the inflammatory state of BM, which is induced by NB disease regardless of the NB infiltration of the BM niche (47). Indeed, previous reports highlighted that inflammation is related to a worse clinical outcome in NB patients (48, 49). Furthermore, tumor cells not only cross-talk with immune and non-immune cells and signal within the primary tumor microenvironment, but neoplastic cells may also generate signals traveling long distances, inducing pre-metastatic niche and promoting the spread of primary tumors (50). Thus, we cannot exclude the presence of EVs in the BM microenvironment could be due to the presence of NB cells in the primary tumor site.

In conclusion, we found evidence supporting the fact that EVs isolated from BM of NB patients may be predominantly secreted by normal resident BM cells. These vesicles, differently from EVs reported in other neoplastic tissues, where they represent an immune mechanism of escape performed by metastatic cells (18, 36, 37, 39–44, 46, 48, 49, 51), can be secreted by normal cells as a mechanism to counteract the inflammation-dependent immune-suppressive state occurring in the marrow of patients with neuroblastoma. However, such a hypothesis requires further investigation and needs to be confirmed in future studies where the inflammatory state of BM-resident cells, as well as the expression of other markers on BM-derived EVs, will be specifically addressed and elucidated.

## Data Availability

The raw data supporting the conclusions of this article will be made available by the authors, without undue reservation.

## Supplementary material

Supplementary Figure 1 Flow cytometric analysis. Representative distribution of EV isolated from one NB patient’s BM sample, and representative plots for isotype controls and staining with anti-CD56, anti-HLA-G, anti-PD-1 and anti-PD-L1 mAbs.

Supplementary Figure 2 Gating strategy for proliferation assay. Cells were firstly gated on lymphocytes, and then on CD3^+^ and CD4^+^ or CD4^-^ cells. CFSE dilution was analyzed on CD4^+^ or CD8^+^ (CD3^+^CD4^-^) T lymphocytes.

Supplementary Figure 3 ROC curve analysis in NB patients. Data from ROC curve analysis performed in NB patients on the expression of HLA-G, CD56, PD-L1 and PD-1. **(A)** shows data obtained by the analysis of EFS, whereas **(B)** shows data obtained with OS.
