# Supplementary figures and images for: Immunomodulatory properties of extracellular vesicles isolated from bone marrow of patients with neuroblastoma: role of PD-L1 and HLA-G
Source: Front Immunol. 2024 Oct 24;15:1469771. doi: 10.3389/fimmu.2024.1469771 (PMC11540764; doi:10.3389/fimmu.2024.1469771)

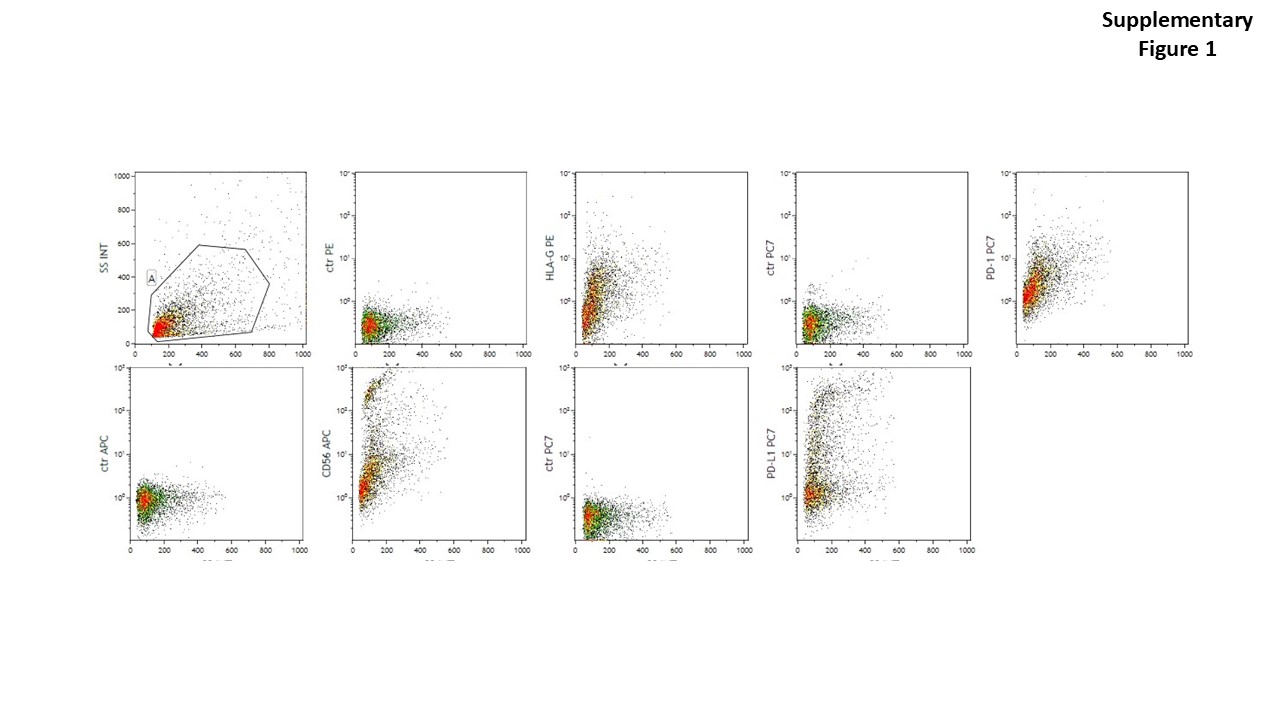

Supplement: Supplementary Figure 1 — Flow cytometric analysis. Representative distribution of EV isolated from one NB patient’s BM sample, and representative plots for isotype controls and staining with anti-CD56, anti-HLA-G, anti-PD-1 and anti-PD-L1 mAbs. [file Image1.jpeg]

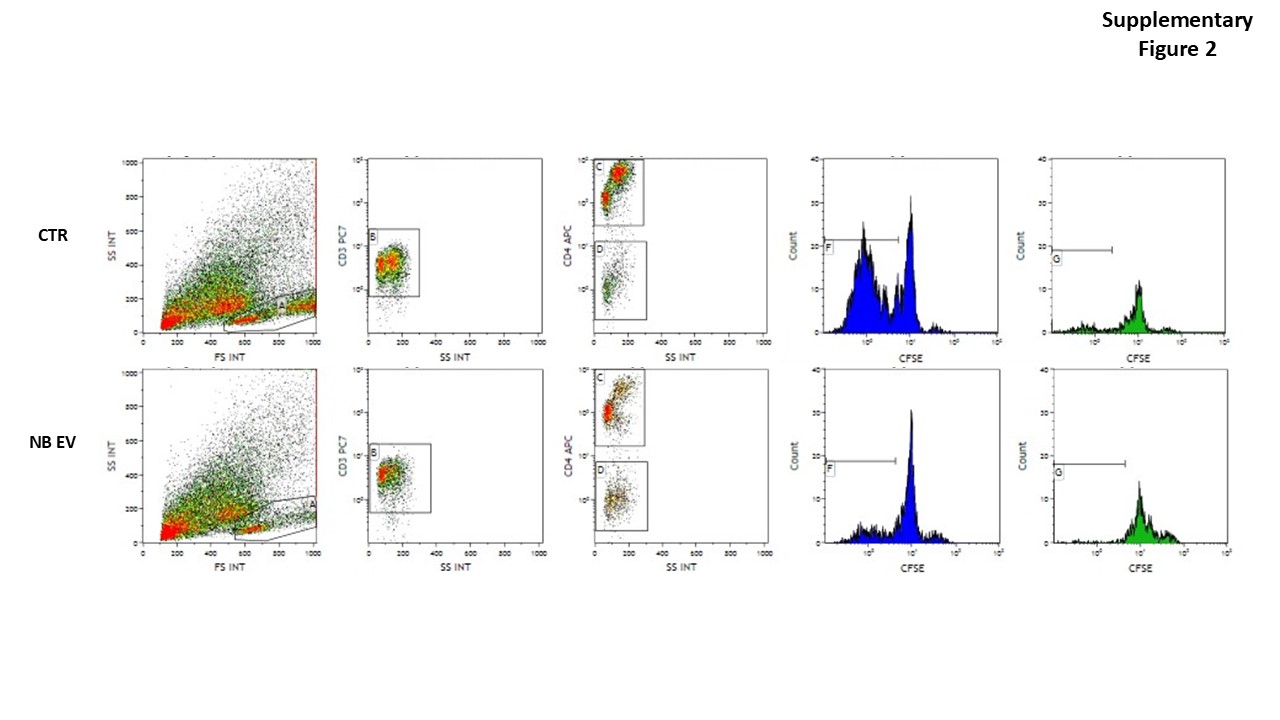

Supplement: Supplementary Figure 2 — Gating strategy for proliferation assay. Cells were firstly gated on lymphocytes, and then on CD3+ and CD4+ or CD4- cells. CFSE dilution was analyzed on CD4+ or CD8+ (CD3+CD4-) T lymphocytes. [file Image2.jpeg]

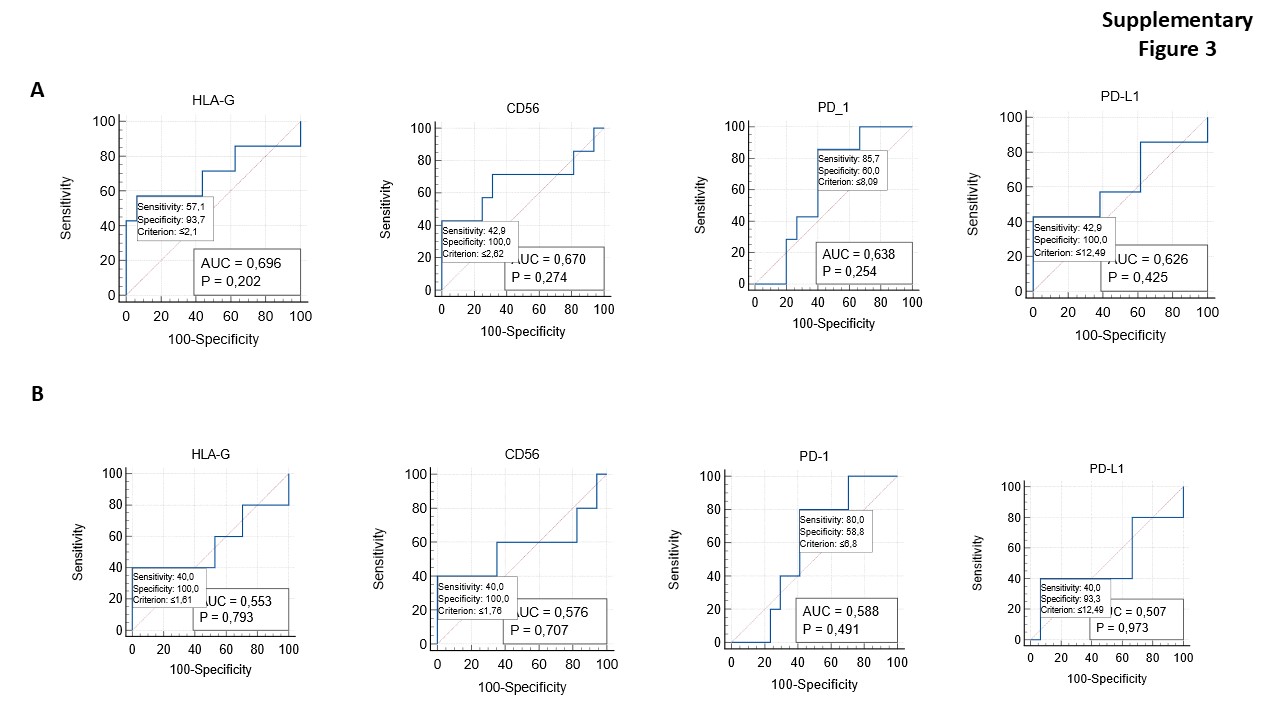

Supplement: Supplementary Figure 3 — ROC curve analysis in NB patients. Data from ROC curve analysis performed in NB patients on the expression of HLA-G, CD56, PD-L1 and PD-1. (A) shows data obtained by the analysis of EFS, whereas (B) shows data obtained with OS. [file Image3.jpeg]
